# An environmental DNA sampling method for aye‐ayes from their feeding traces

**DOI:** 10.1002/ece3.4341

**Published:** 2018-07-31

**Authors:** Megan L. Aylward, Alexis P. Sullivan, George H. Perry, Steig E. Johnson, Edward E. Louis

**Affiliations:** ^1^ Department of Anthropology and Archaeology University of Calgary Calgary AB Canada; ^2^ Department of Biology Pennsylvania State University State College Pennsylvania; ^3^ Department of Anthropology Pennsylvania State University State College Pennsylvania; ^4^ Grewcock Center for Conservation and Research Omaha's Henry Doorly Zoo and Aquarium Omaha Nebraska

**Keywords:** endangered, environmental DNA, lemur, mitogenome, noninvasive sampling, target capture

## Abstract

Noninvasive sampling is an important development in population genetic monitoring of wild animals. Particularly, the collection of environmental DNA (eDNA) which can be collected without needing to encounter the target animal facilitates the genetic analysis of endangered species. One method that has been applied to these sample types is target capture and enrichment which overcomes the issue of high proportions of exogenous (nonhost) DNA from these lower quality samples. We tested whether target capture of mitochondrial DNA from sampled feeding traces of the aye‐aye, an endangered lemur species would yield mitochondrial DNA sequences for population genetic monitoring. We sampled gnawed wood where aye‐ayes excavate wood‐boring insect larvae from trees. We designed RNA probes complementary to the aye‐aye's mitochondrial genome and used these to isolate aye‐aye DNA from other nontarget DNA in these samples. We successfully retrieved six near‐complete mitochondrial genomes from two sites within the aye‐aye's geographic range that had not been sampled previously. Our method demonstrates the application of next‐generation molecular techniques to species of conservation concern. This method can likely be applied to alternative foraged remains to sample endangered species other than aye‐ayes.

## 1 INTRODUCTION

Genetic sampling of wild populations can help us to address questions of demography, individual relatedness, population structure, and other important aspects of biodiversity that cannot be answered by behavioral monitoring alone (Allendorf, Hohenlohe, & Luikart, 2010). However, the practicality of sampling wild animals can be limited by ethical implications due to the risks of sedating animals to collect blood or tissue samples, especially for arboreal species, and for rare or cryptic species (including nocturnal animals), by infrequent encounter rates (Goossens, Chikhi, Utami, de Ruiter, & Bruford, 2000; Kohn & Wayne, 1997). Even when it is safe to collect invasive samples from wild individuals, the number of samples that can be obtained with this approach may be fewer than analytically desirable (Sikes & Gannon, 2011). Under these conditions, noninvasive sampling is a valuable tool in advancing our understanding of wild populations.

Over the last two decades, advances in molecular biology have facilitated the use of noninvasive sampling for genetic analyses of wild populations (Joost et al., 2007; Morin, Wallis, Moore, Chakraborty, & Woodruff, 1993). Furthermore, traces of DNA left in the environment from biological material (eDNA) can be collected without ever encountering target individuals (Taberlet, Bonin, Zinger, & Coissac, 2018). Source materials for noninvasive or eDNA sampling that have been used in wildlife monitoring and forensic studies include feathers, feces, egg shells, hair, saliva, urine, and snail trails (Beja‐Pereira, Oliveira, Alves, Schwartz, & Luikart, 2009; Inoue, Inoue‐Murayama, Takenaka, & Nishida, 2007; Sastre, Francino, Sanchez, & Ramirez, 2009; Valiere & Taberlet, 2000). Noninvasive and eDNA sampling are particularly valuable in research surrounding unhabituated populations, cryptic species, and those at risk of extinction (Clevenger & Sawaya, 2010; Cushman, McKelvey, Hayden, & Schwartz, 2006; Goossens et al., 2000; KendallL et al., 2009; Morin, Kelly, & Waits, 2016; Orkin, Yang, Yang, Yu, & Jiang, 2016; Schubert et al., 2011).

One species that could especially benefit from the advances in noninvasive genetic sampling is the aye‐aye (*Daubentonia madagascariensis*), which is a rare and elusive Malagasy primate of major conservation concern (Schwitzer et al., 2013; Sterling & McCreless, 2007). The low population density, large home range size, cryptic nature, and nocturnal activity of aye‐ayes make them particularly difficult to locate, and precise distributions and population densities are unclear (Sterling, 1994b). Challenges of monitoring aye‐ayes and obtaining reliable population dynamics data are reflected in the volatility of aye‐aye's conservation status designations over the last 70 years. In the 1950s, the aye‐aye was thought to be extinct (Sterling, 1994b). After aye‐ayes were rediscovered in 1957, they were classified as Endangered; in 2008, their status was changed to Near Threatened before being reassessed as Endangered in 2012 (Andriaholinirina et al., 2015). In addition, aye‐ayes are currently considered one of the world's top 25 most endangered primates (Schwitzer et al., 2017). Few encounters, along with the aye‐aye's solitary social organization and long maternal investment suggest low population densities. Low nuclear genomic diversity in aye‐ayes reflects these assumptions; genomic analyses estimates of heterozygosity of 0.051%, and genetic diversity across synonymous sites of *π* = 0.073, are the lowest of any primate species studied to date (Perry, Melsted, et al., 2012; Perry, Reeves et al., 2012). Therefore, despite the wide distribution of aye‐ayes, there are likely few individuals, increasing the risk of local and global extinction (Gross, 2017; Schwitzer et al., 2013).

The IUCN's lemur survival strategy recognizes the need for biological monitoring of aye‐ayes to better assess population status and conserve genetic diversity within this lineage (Schwitzer et al., 2013). In addition to the difficulties in finding and monitoring aye‐ayes, invasive collection of blood and tissue for genetic sampling can only be achieved during immobilization, which is risky and must be conducted by trained and experienced personnel (Cunningham, Unwin, & Setchell, 2015). Therefore, to assess genetic diversity and identify priority populations for conservation a new, reliable means of noninvasive sampling in aye‐ayes is required.

Reliable genotyping from noninvasively collected material holds much promise in sampling threatened species. Environmental DNA samples that are degraded due to exposure to both biotic and abiotic factors or contain high proportions of exogenous DNA can now provide reliable genetic markers (Beja‐Pereira et al., 2009; Carpenter et al., 2013; Snyder‐mackler et al., 2016). Techniques such as high‐throughput sequencing technologies reduce sequencing errors and improve genotyping accuracy by providing greater depth of coverage across loci. One particularly promising approach is target capture, which provides a means of isolating endogenous DNA from the high proportions of exogenous DNA in noninvasive samples (Bi, Linderoth, Vanderpool, Good, & Nielsen, 2013; Hawkins et al., 2015; Kirillova et al., 2015; Kistler et al., 2015; Mohandesan et al., 2017). Specifically, RNA probes which are complementary to particular regions or markers in the genome of the target organism are specially designed and synthesized (Gnirke et al., 2009). These probes hybridize to the target DNA in the sample library. After hybridization, the biotin coating of the probes allows streptavidin‐coated magnetic beads to bind; the bound probes and hybridized endogenous DNA can then be isolated from the exogenous DNA using a magnet (Giolai et al., 2016; Gnirke et al., 2009). Compared to conventional PCR for amplification, target capture and NGS may be more effective for degraded, noninvasive, or eDNA samples as multiple short fragments of DNA are captured and sequenced, and ultimately larger regions of DNA can be targeted (Gnirke et al., 2009). These developments make monitoring and sampling of wild populations where individuals are difficult to locate increasingly feasible.

For primates, the application of target capture from low‐quality samples has largely been applied to ancient DNA studies, but Perry, Marioni, Melsted, and Gilad (2010) and Snyder‐mackler et al. (2016) have presented effective approaches for sampling endogenous DNA from feces in extant primate species. Recently, Chiou and Bergey (2018) demonstrated a different method that isolates endogenous DNA from primate feces by targeting the higher CpG‐methylation density of vertebrate taxa relative to the exogenous bacterial and plant DNA in feces. Although fecal collection is a popular source of noninvasive sampling of primates (Chancellor et al., 2011; Oka & Takenaka, 2001; Quéméré, Crouau‐Roy, Rabarivola, Louis, & Chikhi, 2010), it is unlikely to be a feasible method of sampling for aye‐ayes. Factors such as aye‐aye's nocturnal behavior, the height at which they travel in the canopy, and their large nightly travel distances mean that defecation is difficult to observe and locating and collecting fecal material is problematic (Randimbiharinirina et al., 2017); accordingly, at two sites where aye‐ayes have been monitored by Madagascar Biodiversity Partnership since 2010, aye‐aye fecal samples have only been collected through routine immobilizations. Therefore, to gain information on the genetics of aye‐aye populations to meet the IUCN aims (Schwitzer et al., 2013), we explore the possibility of sampling eDNA from aye‐ayes.

One potential source of eDNA in aye‐ayes is from their distinct feeding traces left on trees. These traces are associated with their adaptations for extracting the larvae of wood‐boring insects from tree trunks and branches, after identifying suitable foraging locations via a process of sniffing, lightly tapping, and listening (Erickson, 1995; Sterling, 1994a). Aye‐ayes gnaw into selected areas of trees with their elongated and continuously growing incisors and extract larvae using their thin flexible third digit (Sterling, 1994a). During this foraging process, the buccal cavity of the aye‐aye comes into contact with the wood (Erickson, 1995; Sterling, 1994a; Sterling & McCreless, 2007). Thus, trace amounts of aye‐aye biological material in the form of epithelial cells of the buccal mucosa or from saliva may be deposited and accessible as an eDNA source.

We investigated the application of target capture and enrichment to obtain aye‐aye DNA from aye‐aye feeding traces to determine whether this method is a feasible alternative to invasive sampling. If eDNA samples provide a means of remotely sampling wild aye‐aye populations, we predict that (a) target enrichment is an effective method of obtaining aye‐aye DNA from the exogenous DNA in feeding traces; and (b) we will be able to obtain full mitochondrial genomes (hereafter “mitogenomes”) for population genomic analysis.

## 2 METHODS

### 2.1 Sampling sites and techniques

Initial training for researchers and local technicians on accurate identification of aye‐aye feeding traces and the sample collection method was conducted through the Kianjavato Ahmanson Field Station (KAFS) located in the Kianjavato commune of the Vatovavy‐Fitovinany region in south‐east Madagascar during June 2014. KAFS is a long‐term field site of the Madagascar Biodiversity Partnership (MBP). Individual aye‐ayes present in the region have been fitted with ATS (Applied Telemetry Systems^®^) VHS radio‐collars for behavioral monitoring, which afforded the opportunity to view known feeding sites in an area of bamboo to become familiar with density and frequency of feeding traces, their characteristics, and to practice the collection technique (see below).

**Figure 1:**
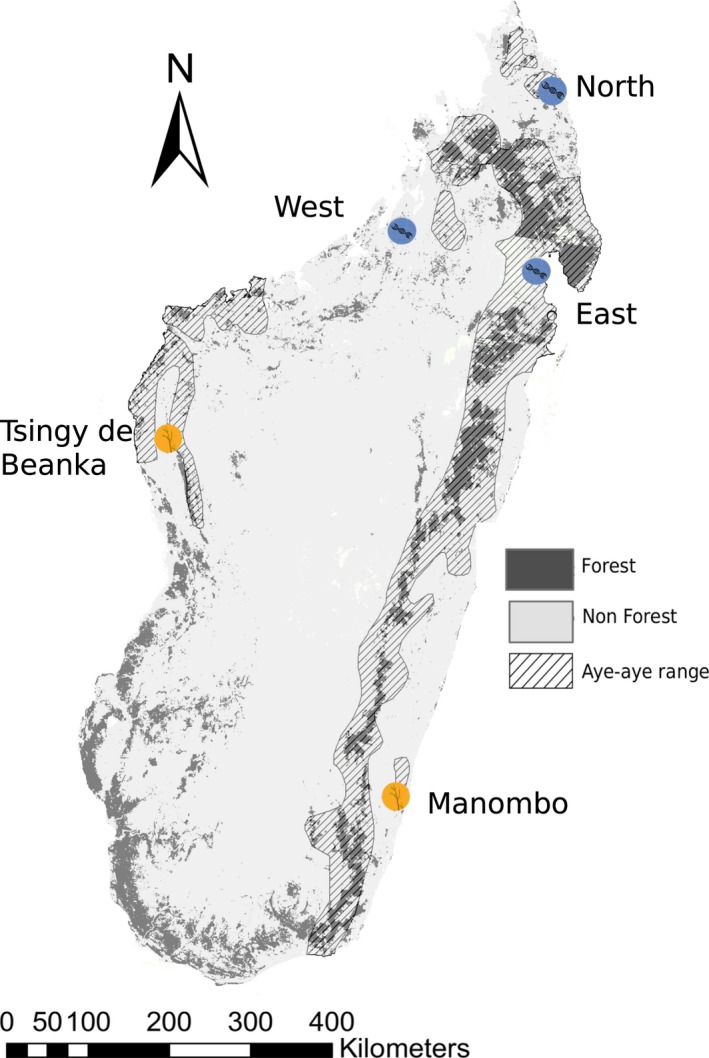
A map of the island of Madagascar showing sample locations targeted in this study. The orange circles highlight the two locations surveyed for aye‐aye feeding traces and eDNA samples were collected. The blue circles indicate the locations that were sampled in Perry et al., 2013; from which the mitogenome sequences from Kistler et al., 2015 were sampled

**Figure 2:**
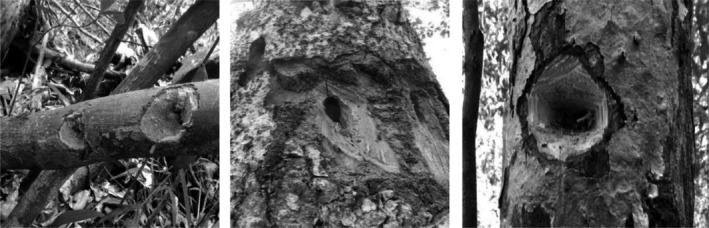
Aye‐aye feeding traces. These images indicate the typical aye‐aye feeding traces showing distinct shapes and teeth marks. These traces centre on a hole where the aye‐aye has used its specialized extractive foraging strategy to access wood boring insect larvae. We sampled pieces of wood around the edge of the trace where the buccal cavity of the aye‐aye comes into contact with the tree

To then test the sampling method on unmonitored populations, we selected two study sites near to the limits of the aye‐aye's geographic distribution, where aye‐ayes have been sighted but not sampled: Manombo Special Reserve, a rainforest environment, in the Atsimo‐Atsinanana region in south‐eastern Madagascar, operated by Madagascar National Parks and at that time supported by Durrell Wildlife Conservation Trust, and the Tsingy de Beanka, Ambinda, a dry deciduous forest in the Melaky region in west‐central Madagascar and supported by Biodiversity Conservation Madagascar (Figure 1). Between July and December 2014, MA surveyed the forest at each site with the assistance of three local technicians. Prior to sampling, local technicians cleared undergrowth from existing trail systems in the forest to use as transects. During daylight hours, these transects were walked at a slow pace of approximately 1 km/hr to look for feeding traces and searched areas on either side of the transect at 200 m intervals. Traces were observed in both live and dead plants where characteristic holes gnawed by aye‐aye were identified. These were confirmed by the distinct shape of feeding traces and typically presence of teeth marks (Figure 2). The research permit (No. 162/14/MEF/SG/DGF/DCB.SAP/SCB) for sample collection was obtained from the Ministère de l'environnement, des eaux et forêts et du tourisme, in Madagascar.

To collect samples, we selected areas around the edge of the trace where the buccal cavity and teeth of the animal were likely to have come into contact with the tree (Figure 2). MA took 3–6 samples of tree material per trace; each sample was approximately 2 g of wood shavings. MA collected samples using a scalpel to cut wood from the site directly into a 1.5 ml Eppendorf^®^ tube containing 500 μl RNAlater. To reduce the chance of contamination, MA wore latex gloves and used a new, sterile scalpel blade for each sample. At Manombo, we walked 9.5 km of transects over a 6 km^2^ area. At Beanka, we surveyed 10.8 km of transects covering a total 9 km^2^ area. MA sampled a total of 59 traces across the two sites (Manombo: *n* = 27; Beanka: *n* = 32). We stored samples at ambient temperature until they could be transferred to 4°C for storage in Antananarivo, Madagascar. MBP exported samples from Madagascar (Export Permit Number 233N‐EV07/MG16) and stored the samples at Omaha's Henry Doorly Zoo and Aquarium at 4°C for 4 months prior to processing.

### 2.2 DNA extraction, library preparation, capture, and sequencing

After samples were removed from storage, we extracted DNA from all samples. We vortexed each sample for 30 s in PBS to wash aye‐aye DNA from the surface of the wood and then removed pieces of wood using sterile forceps. We added 500 μl of PBS solution to the sample and centrifuged at 10,000 *g* to pellet the cellular DNA and then carefully removed the supernatant so as not to disturb the precipitate. We performed all DNA extractions on the precipitate using EZNA^®^ blood DNA mini kit following the buccal swab protocol with the following modifications: we lysed samples overnight, and we eluted DNA in 50 μl elution buffer, incubated for 20 min and then re‐eluted with the same eluate. Preliminary tests to confirm the presence of aye‐aye DNA were conducted using a microsatellite marker for loci AYE33 of approximately 279 bp, with the following primer pair: forward 5′‐3′ GTCTGCTACTCCTTAGGTGCTG and reverse 5′‐TGGCTCAGGGCAATACAAT‐3′. We amplified the extracted DNA in a 15 μl reaction containing 0.25 μl of each 10 μM primer, 7.5 μl buffer containing 1.5 mM MgCl_2_ (1X) and 0.2 mM of each dNTP (1X), 0.12 μl KAPA3G plant DNA polymerase, 4.9 μl H_2_O, and 2 μl DNA sample. Cycling parameters began with an initial enzyme activation temperature of 95°C for 3 min, followed by 32 cycles of denaturation at 98°C for 20 s, primer annealing at 58°C for 15 s and elongation at 72°C for 30 s, then one cycle of final elongation at 72°C for 1 min. We visually confirmed the presence of the expected 279 bp PCR product by gel electrophoresis with 5 μl of PCR product and using 2 μl of a 1 kb ladder.

Extracted DNA was transported to Pennsylvania State University, University Park, PA for library preparation and DNA capture. We quantified the DNA concentration and fragment size of ten samples using a bioanalyzer (Agilent Technologies, Santa Clara, CA) at the Huck Institute of the Life Sciences (HILS), University Park, PA, genomics core facility. We selected a subset of 22 samples that were estimated to have been between 14 hrs and 6 months old at the time of collection based on observations during surveys and discolouration of wood. We prepared DNA sequencing libraries from each of these samples using the Illumina TruSeq nano^®^ kit. The TruSeq nano^®^ library preparation protocol recommends 200 ng of input DNA. Due to the typically low quantities of DNA extracted from each individual wood sample (<300 pg/μl), we combined DNA extracted from multiple samples taken from the same trace into one sample per feeding trace to increase total amounts of library preparation input DNA per sample to approximately 5–42 ng DNA (based on bioanalyzer readings for a subset of samples). To account for the low input DNA quantities, we adapted the protocol to use half quantities of all reagents and volumes. We sheared each DNA sample to a mean size of 500 bp with the Covaris‐M220^®^ using the following settings: 34 s, peak incident of 50, duty factor of 20% and 200 cycles per burst. For library preparation wash steps, 180 μl ethanol was used and an additional initial wash step was included to remove residual salts from eluting extracted DNA. We used the Bioanalyzer (Agilent Technologies) to quantify the final library and confirm a mean library size of 750 bp fragments, as expected for 550 bp libraries once the sequencing adapters and unique barcodes are attached to the 550 bp DNA fragments.

As a preliminary test to check for the presence of aye‐aye DNA in these libraries, we shotgun sequenced ten DNA libraries. Libraries were included as part of a larger multiplexed sequencing pool (Supporting Information Table S1) and sequenced using the Illumina NextSeq^®^ 500 2 × 150 pair end chemistry at UCLA Clinical Microarray Core Facility. Samples were demultiplexed at the sequencing facility.

RNA probes for capture of the aye‐aye mitogenome were designed through MyBaits^®^ at Arbor Biosciences^™^. Baits were designed based on the aye‐aye reference mitochondrial DNA genome sequence (GenBank^®^ accession NC_010299.1), bait length was 80 bp at 4x tiling and these are available as a predesigned MyBaits^®^ Mito panel. We conducted captures as per MyBaits^®^ manual v.3 and a single round of captures was conducted on 19 of the eDNA (foraging remains) samples. Postcapture products were quantified using Bioanalyzer (Agilent Technologies) at the genomics core facility at the HILS and pooled for sequencing (Supporting Information Table S2). These capture products were sequenced as one pool of 17 samples and two of the samples (BTS38 and BTS108) were included as part of a different sequencing pool. The final mitochondrial capture pool of 17 Mito Bait captured DNA libraries was sequenced on one lane of the NextSeq 500 mid‐output 2 × 150 bp pair end chemistry UCLA Clinical Microarray Core Facility. Samples were demultiplexed and barcodes removed at the sequencing facility.

### 2.3 Data analysis

Raw reads were submitted to the NCBI SRA project under the accession PRJNA434884. All computational analyses were conducted on servers provided by WestGrid (http://www.westgrid.ca) at Compute Canada (http://www.computecanada.ca). The following processing and alignment pipeline was used for the shotgun and captured DNA sequencing pools. We assessed sequence read quality using Fast‐QC (Andrews, 2007) and filtered reads by selecting those with a minimum read length of 120 bp and mean Phred scaled quality score of 20 using PRINSEQ‐lite (Schmieder & Edwards, 2011). We removed any potential human contaminant reads using the bbsplit package of bbmap (Bushnell, 2016); sequencing reads were aligned to both the aye‐aye and human genome and any reads which matched better to the human genome than the aye‐aye genome were removed from the dataset. Paired reads were aligned to the aye‐aye reference genome GCA_000241425.1 (Perry, Reeves, et al., 2012), using the bwa‐mem alignment algorithm (Li, 2013; Li & Durbin, 2009). We used the MarkDuplicates tool in PicardToolsv.1106 (Broad Institute, 2017) to mark and remove sequencing and PCR duplicates, and reads flagged as clipped were filtered from the dataset. We generated consensus sequences from the unique read data for the eDNA samples with near‐complete mitogenomes using samtoolsv.1.3 mpileup command, bcftoolsv.1.3 ‐call and vcfUtilsv.1.3 (Danecek et al., 2011; Li, 2011; Li et al., 2009), where ploidy was set to 1 and the most evident nucleotides are kept (Li, 2011). To confirm the base calls in these consensus sequences, we compared the samtoolsv.1.3 generated sequences to those generated using IGV (Robinson et al., 2012) to ensure the same nucleotide bases were found by each software program.

We compared the mitogenome sequences from our eDNA samples with the GenBank^®^ aye‐aye mitogenome data to confirm that they were novel (Kistler et al., 2015; Perry et al., 2013). We called variants using GATK (McKenna et al., 2010), and used haplotype caller to generate individual gVCFs and then the joint variant caller. To filter for biallelic SNPs only, we used VCFtoolsv.1.12 and set maximum and minimum alleles to two.

We used PGDSpiderv2.3 to convert SNP variant calls from vcf to ped format (Lischer & Excoffier, 2012). We used the—cluster and—matrix commands in Plinkv.1.7 (Purcell et al., 2007) to calculate the pairwise proportion (identity by state, IBS) of shared alleles at SNP loci among all complete mitogenome sequences. We estimated a phylogenetic tree using MCMC approach implemented in BEASTv2.4.6 (Bouckaert et al., 2014). We implemented a clock rate of 1.113 × 10^−8^ substitutions/site/year and substitution rate was estimated using bmodel test (Bouckaert & Drummond, 2017).

## 3 RESULTS

### 3.1 Precapture processing

After library preparation, shotgun sequencing of libraries from eDNA samples did not yield sufficient mitochondrial DNA for mitogenome analysis. Of the 10 libraries that were shotgun sequenced, seven yielded fewer than 10 reads aligning to the aye‐aye mitochondrial genome; one sample, with the highest number of mitochondrial reads, was MSR58 with 189 unique reads (0.000274%; Table 1).

**Table 1:** The fold increase in percent of unique on‐target reads aligning to the aye‐aye mitochondrial genome target MitoBait captures compared to shotgun sequencing of the DNA libraries. Data show for the ten libraries that were shotgun sequenced prior to capture

Sample ID	Number of on‐target reads	Unique on‐target reads, shotgun sequencing (%)	Unique on‐target reads, from captured DNA libraries (%)	Fold enrichment
MSR01	0	0.000000	0.0027	—
MSR44	8	0.000038	0.0538	1,416
MSR46	0	0.000000	0.0019	—
MSR50	31	0.000063	0.0248	394
MSR58	189	0.000274	0.0408	149
BTS04	0	0.000000	0.0051	—
BTS38	6	0.000103	0.0267	259
BTS60	0	0.000000	0.0005	—
BTS108	24	0.000124	0.0139	112
BTS112	0	0.000000	0.0056	—

### 3.2 Target capture DNA sequencing

Target captures of the mitogenome increased the proportion of on‐target mitochondrial DNA reads by several orders of magnitude compared to reads yielded from shotgun sequencing without capture (Table 1). For samples for which it was possible to calculate the fold enrichment (i.e., the samples that were both shotgun sequenced and sequenced following DNA capture), the percent of unique on‐target reads increased by over 100 times. For captured samples, the percent of on‐target reads ranged from 0.009% to 27.5% of the total reads, whereas the percent unique on‐target reads ranged from 0.001% to 0.05% (Table 2).

**Table 2:** Sequencing summary statistics showing number of reads and depth and breadth of coverages for MitoBait captures. * indicates samples with over 90% breadth of coverage that were used for comparisons with published mitogenomes

Sample site	Sample ID	Reads on‐target (%)	Unique reads on‐target (%)	Mean read depth (*SD*)	Mean % coverage
Tsingy de Beanka	BTS04	0.06	0.0015	1.56 (0.9)	59.3
**Tsingy de Beanka**	BTS27*	0.38*	0.0063*	13.0 (5.3)*	99.4*
**Tsingy de Beanka**	BTS38*	0.22*	0.027*	23.3 (5.9)*	98.0*
Tsingy de Beanka	BTS60	0.02	0.0005	1.8 (2.2)	45.6
**Tsingy de Beanka**	BTS68*	4.3*	0.0088*	8.5 (6.0)*	98.9*
Tsingy de Beanka	BTS102	0.26	0.0013	1.8 (1.4)	27.3
Tsingy de Beanka	BTS105	0.5	0.0005	1.7 (1.2)	22.1
**Tsingy de Beanka**	BTS108*	0.4*	0.014*	15.1 (5.6)*	99.5*
Tsingy de Beanka	BTS112	0.46	0.0056	1.3 (0.6)	10.9
Manombo Special Reserve	MSR01	0.05	0.0027	2.0 (1.4)	65.9
Manombo Special Reserve	MSR23	0.04	0.039	1.2 (0.4)	12.3
Manombo Special Reserve	MSR37	0.16	0.0002	1.1 (0.3)	11.1
Manombo Special Reserve	MSR39	27.54	0.0024	5.0 (11.83)	45.5
Manombo Special Reserve	MSR44	22.25	0.05	1.5 (1.2)	21.9
Manombo Special Reserve	MSR46	4.11	0.0019	1.3 (0.5)	10.8
Manombo Special Reserve	MSR50	12.19	0.025	2.5 (2.07)	70.9
Manombo Special Reserve	MSR56	0.024	0.0001	1.3 (0.6)	15.0
**Manombo Special Reserve**	MSR58*	2.71*	0.04*	12.4 (7.2)*	98.5*
**Manombo Special Reserve**	MSR62*	2.20*	0.015*	25.1 (8.8)*	99.8*

We recovered near‐complete mitogenomes for six of the 19 captured samples (31.6%). The depth and breadth of coverage varied among samples (Table 2). For these six mitogenomes, the mean depth of coverage was 16x (range 8–25x). For the 13 samples with <90% coverage, the percent of the genome covered ranged from 10%–71% (mean 32.58%) with a mean depth of 1.7 (±0.3–2.8). Five of these 13 samples had a breadth of coverage between 45%–70% and were all identical at SNP loci to the samples with greater breadth of coverage. We only included samples with coverage >90% in downstream analysis.

### 3.3 Mitogenome analysis

**Figure 3:**
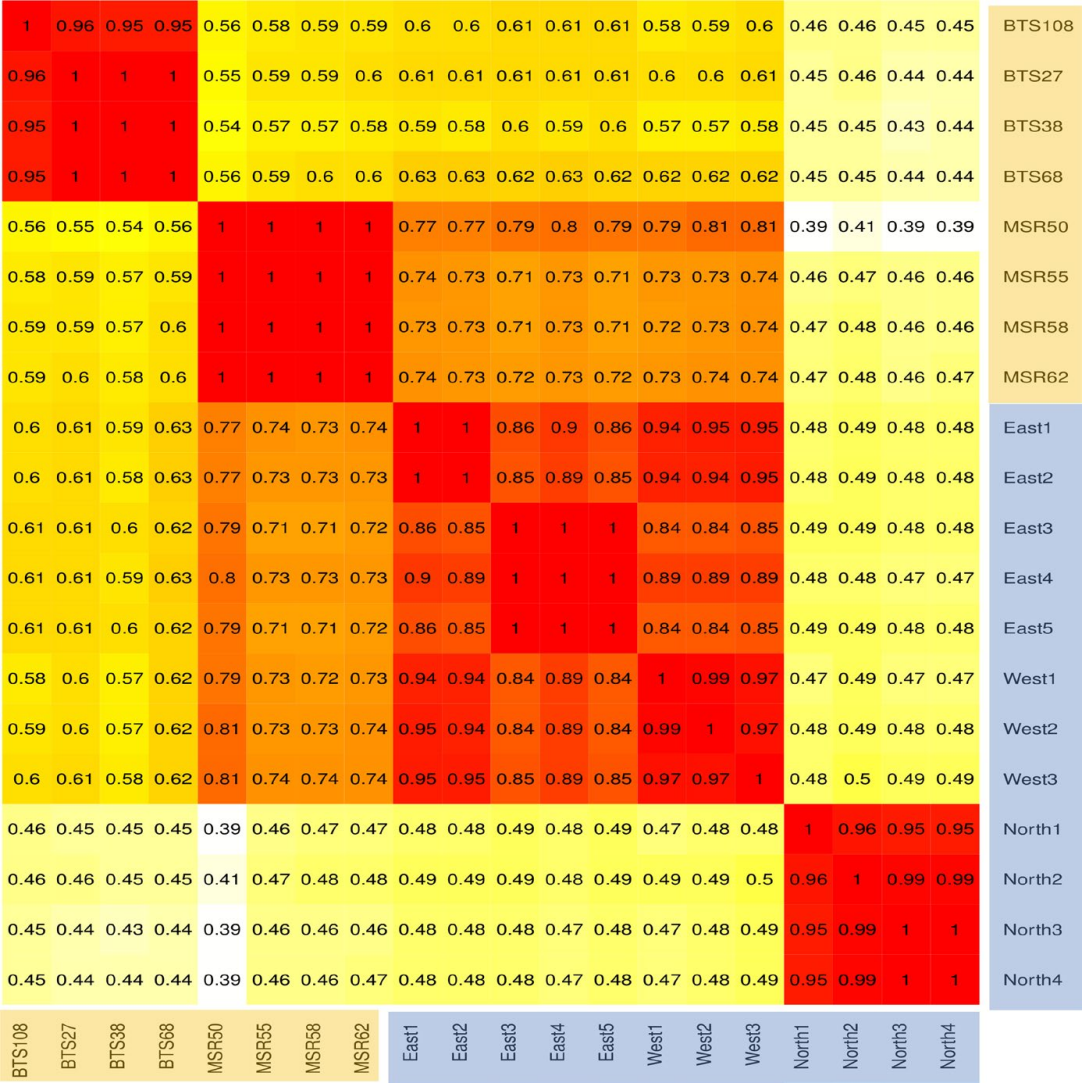
Identity by state matrix which shows the proportion of pairwise shared alleles at each SNP site for all polymorphic loci across the mitogenomes. We compared eDNA samples from feeding traces (shaded in orange) with previously published mitogenome sequences (Kistler et al., 2015) shaded in blue. Warmer matrix colours indicate greater proportions of shared alleles at these loci

Based on multiple sequence alignment of these seven near‐complete mitogenomes, we identified one unique mitogenome sequence from Manombo Special Reserve and two unique mitogenomes from Tsingy de Beanka. Alignment with previously published GenBank^®^ sequences showed that the mitogenome sequences obtained from the eDNA samples were unique to all previously published aye‐aye mitogenomes. For the GenBank sequences sample locations from northern Madagascar, we follow the population names in Perry et al. (2013); North, East, and West (Figure 1). When we compared SNP loci among samples, all mitogenomes clustered into distinct populations according to their collection location (Figure 3). Pairwise comparison of genotypes at polymorphic loci indicate that there are four distinct populations which correspond to sampling locations, Tsingy de Beanka, and Manombo from our eDNA sampling, the North, and East and West populations sampled in Perry et al. (2013) (Figure 3). The Tsingy de Beanka population is divergent from the other populations (Figure 3). There was also a split between the East population and Manombo in the southeast, while the North population shared the fewest number of SNPs with all other populations. The East and West populations are not clearly resolved based on mitogenome sequences; two of the East individuals share greatest proportions of alleles with the West population, and all East and West individuals share over 85% of alleles. These relationships were reflected in the phylogenetic tree, whereby each population is divergent, with the exception of the East and West populations defined in Perry et al. (2013), where two of the East individuals formed a sister clade with the West individuals (See Supporting Information Figure S1).

## 4 DISCUSSION

We tested whether sampling of aye‐aye feeding traces is a feasible method of noninvasive sampling of this species. Our first prediction, that target capture would provide a means of sampling aye‐aye DNA from these feeding traces, was supported by the increased amount of endogenous DNA obtained from target capture compared to shotgun sequencing. The number of on‐target reads from the shotgun sequencing was not at sufficient coverage across the mitogenome to identify polymorphic sites and genotype individuals. The high level of enrichment from captured libraries compared to shotgun sequencing indicates the efficacy of a target capture approach (Gnirke et al., 2009). Our second prediction, that mitogenomes could be obtained from these samples and used for population genomic analysis, was also supported. We were able to obtain near complete mitogenomes for 31.6% of the processed samples. Previous studies that have sampled eDNA from foraged material have typically only retrieved partial D‐loop fragments rather than whole mitogenomes (Caniglia, Fabbri, Mastrogiuseppe, & Randi, 2013; Nichols, Konigsson, Danell, & Spong, 2012; Wheat, Allen, Miller, Wilmers, & Levi, 2016). While partial fragments can be useful in metabarcoding studies for species detection, whole mitogenomes provide greater phylogenetic resolution and reduce the likelihood of including nuclear copies of mitochondrial DNA (numts) (Johnsen, Kearns, Omland, & Anmarkrud, 2017).

### 4.1 Future directions for methodological improvements for sampling aye‐aye eDNA

The method developed here is a valuable tool to extend sampling of unmonitored aye‐aye populations. Yet, improved efficacy of the method to increase amount of aye‐aye DNA obtained and therefore reduce the cost per sample could make it a more attractive application for conservation monitoring (Ekblom & Galindo, 2010). Low proportions of on‐target, unique sequence reads recovered are common when working with environmental samples (Ávila‐Arcos et al., 2015; Nielsen et al., 2017) and adequate sampling effort is required to account for this issue. A mode of three different samples was taken per feeding trace, and it would be feasible to double this number. Therefore, we recommend more intensive sampling of each feeding trace to allow for multiple library preparations per trace, while maintaining sufficient amounts of input DNA for TruSeq nano^®^ library preparation. Multiple library preparations for each trace prior to capture may increase the number of unique DNA fragments within the samples and reduce PCR bias. The proportion of unique reads aligning to the target region was relatively low (0.4%–12%). To better assess the number of PCR cycles to run postcapture to achieve sufficient concentrations for sequencing, qPCR quantification may be valuable to ensure reduced PCR duplication, therefore improved sequencing efficacy and ultimately increased cost effectiveness (Enk, Rouillard, & Poinar, 2013).

### 4.2 Application toward addressing the IUCN's objectives for aye‐ayes

We demonstrated a novel method that can be applied across the species range for population mitogenomic monitoring. This method helps to meet objective seven of the IUCN's lemur survival plan set out by Schwitzer et al. (2013, 32): “Fill knowledge gaps in population ecology and biodiversity of lemurs.” Our method provides a means of mitogenomic biodiversity monitoring using cutting‐edge molecular techniques. Application of this method allowed us to successfully recover six aye‐aye mitogenomes from sites in the southeast and west of Madagascar where aye‐ayes have not been sampled previously. The wide distribution of aye‐ayes means sampling across environments is key in effective population monitoring at local and national scales (Schwitzer et al., 2013). The different habitat types range from rainforest in southeast Madagascar, a habitat with relatively high humidity and rainfall, to the dry deciduous forest in the west of Madagascar, with less dense canopy cover and likely greater exposure to direct UV radiation (Andriamisedra, Aylward, Johnson, Louis, & Raharivololona, 2015; Rakoto‐Joseph, Garde, David, Adelard, & Randriamanantany, 2009). Our recovery of mitogenomes from both environments indicates these abiotic factors do not necessarily preclude the ability to sample eDNA from these areas and this method can likely be applied to sample in varied habitats across the species’ geographic distribution (Sterling & McCreless, 2007). The mobility of this sampling technique along with little training required for sample collection provides a means of surveying large areas of aye‐aye habitat in a relatively short time frame. The ease of sampling and ability to survey large areas provide the opportunity for sampling multiple individuals across a population and accessing mitogenome diversity within forest fragments.

Our preliminary analysis revealed high differentiation between the North population and all other sampled locations, consistent with the whole genome analysis of Perry et al. (2013). However, our mitogenome analysis did not resolve the East and West populations from the north of the island to two distinct populations. Although the exact sampling location of these East samples from Perry et al. (2013) is not known; some of these individuals were from a reintroduced population on the island of Nosy‐Mangabe, which may account for the observed paraphyly of the East and West populations. Given that our relatively sparse sampling strategy revealed two genetically distinct populations, we encourage the application of this method and the use of the predesigned aye‐aye Mito panel from MyBaits^®^ to sample additional areas of aye‐aye habitat.

### 4.3 Applications to other species

The aye‐aye is arguably one of the most elusive lemur species in Madagascar (Schwitzer et al., 2013), and other endangered species that are difficult to locate could also benefit from eDNA sampling. The Critically Endangered *Prolemur simus* leave traces on bamboo plants which are similar to those of sympatric bamboo lemurs that feed on the same plant species (Ravaloharimanitra et al., 2011; Tan, 1999). Application of the method presented here could confirm the presence of this threatened species which are typically sparsely distributed and difficult to locate in the wild (Frasier et al., 2015; Ravaloharimanitra et al., 2011; Wright et al., 2008). Similarly, Critically Endangered *Eulemur cinereiceps* could be sampled via traces on gnawed fronds of *Cecropia peltata* trees (Ralainasolo, Ratsimbazafy, & Stevens, 2006). Although the method developed here is novel, sampling of saliva from foraged material has been used previously as a means of noninvasive sampling; gorillas and golden monkeys discard plant material (Smiley et al., 2010), ungulates leave saliva traces on foraged twigs (Nichols et al., 2012), and large carnivores leave saliva on prey (Blejwas, Williams, Shin, Dale, & Jaeger, 2006; Caniglia et al., 2013; Glen et al., 2010; Sundqvist, Ellegren, & Vila, 2008; Wheat et al., 2016). This method of target capture could be applied to these sources to sample mitogenome eDNA from a range of species.

### 4.4 NGS molecular techniques and conservation

This method provides an example of the utility of next‐generation molecular techniques for noninvasive samples toward sampling wild populations in a species that requires conservation attention. The application of target capture and enrichment has been used previously in nonhuman primates to sample fecal and ancient DNA (Kistler et al., 2015; Perry et al., 2010; Snyder‐mackler et al., 2016). We demonstrated the application of similar molecular techniques to sample from an endangered lemur species, revealing broad‐scale population structure across the aye‐aye's geographic range. One area of discussion that arises from reviews of the application of next‐generation molecular techniques to conservation is often the necessity of these novel techniques over more conventional approaches, given the increased cost and amount of data generated (Allendorf et al., 2010; Mcmahon, Teeling, & Höglund, 2014; Shafer et al., 2015). Here, we present both a unique challenge and solution to sampling a low‐density, elusive, and endangered species. These methodological developments are valuable tools that have enabled us to sample and monitor a cryptic species that otherwise has limited genomic sampling potential. As the field of conservation genomics expands methods such as the one presented here can be applied to achieve direct conservation action.

## CONFLICT OF INTEREST

None declared.

## AUTHOR CONTRIBUTIONS

EL, GP, MA, and SJ conceived idea and designed methodology; MA collected samples; MA and AS conducted laboratory work. All authors contributed critically to drafts and gave final approval for publication.

## DATA ACCESSIBILITY

Raw sequence reads have been submitted to GenBank SRA under the accession number SRP133213.
