# Supplementary material for: An environmental DNA sampling method for aye‐ayes from their feeding traces
Source: Ecol Evol. 2018 Jul 31;8(18):9229–40. doi: 10.1002/ece3.4341 (PMC6194247; doi:10.1002/ece3.4341)
Supplement: Supplementary file 1 [file ECE3-8-9229-s001.docx]

**FigS1. Phylogenetic tree estimated using MCMC approach in BEASTv2.4.6 with the near**

**complete mitochondrial genomes obtained from eDNA sampling and sequence data from**

**Kistler et al. (2015).**

**Table S1. Sequencing pool parameters for shotgun sequencing of eDNA libraries.**

| **Sample** | **Conc. ng/ul** | **Vol. in pool** | **Total ng** | **% pool by amount** |
| --- | --- | --- | --- | --- |
| MSR01 | 0.26 | 2 | 0.528 | 6.81 |
| MSR44 | 0.17 | 5.88 | 0.999 | 6.58 |
| MSR46 | 0.46 | 2.17 | 0.999 | 6.58 |
| MSR50 | 0.39 | 2.56 | 0.998 | 6.58 |
| MSR58 | 0.96 | 1.04 | 0.994 | 6.58 |
| BTS04 | 0.53 | 1.89 | 1 | 6.6 |
| BTS38 | 0.03 | 1 | 0.03 | 0.2 |
| BTS60 | 0.70 | 1.43 | 1 | 6.59 |
| BTS108 | 0.20 | 1 | 0.2 | 1.32 |
| BTS112 | 0.34 | 2.94 | 0.999 | 6.58 |
|  |  |  |  |  |

**Table S2. Sequencing pool parameters for MitoBait captures from eDNA samples.**

| **Sample** | **Conc. ng/ul** | **Vol. in pool** | **ng total** | **% pool by amount** |
| --- | --- | --- | --- | --- |
| BTS04 | 0.36 | 11 | 3.96 | 6.7 |
| BTS27 | 6.8 | 3 | 4.08 | 6.9 |
| BTS60 | 0.515 | 7.8 | 4.02 | 6.8 |
| BTS68 | 0.633 | 6.3 | 3.99 | 6.75 |
| BTS102 | 4.4 | 0.8 | 3.52 | 5.96 |
| BTS105 | 1.56 | 2.6 | 4.06 | 6.87 |
| BTS112 | 0.23 | 16.7 | 2.87 | 4.86 |
| MSR01 | 0.43 | 9.4 | 4 | 6.77 |
| MSR23 | 0.546 | 7.3 | 3.99 | 6.75 |
| MSR37 | 0.389 | 10.3 | 4.01 | 6.79 |
| MSR39 | 0.848 | 4.7 | 3.9 | 6.75 |
| MSR44 | 0.003 | 10 | 0.03 | 0.05 |
| MSR46 | 0.003 | 10 | 0.03 | 0.05 |
| MSR50 | 0.0 | 10 | 0 | 0.00 |
| MSR56 | 0.932 | 4.3 | 4.01 | 6.79 |
| MSR58 | 0.209 | 10 | 2.09 | 3.54 |
| MSR62 | 2.66 | 1.5 | 3.99 | 6.75 |
| BTS108 | 0.42 | 6 | 2.5 | N/A |
| BTS38 | 2.2 | 1.8 | 3.96 | N/A |
